# Effect of human survivin‐2B‐specific cytotoxic CD8+ T lymphocytes on CD44+/− HSC‐2 and HSC‐3 oral cancer cells

**DOI:** 10.1111/eos.70019

**Published:** 2025-05-21

**Authors:** Sho Miyamoto, Azuna Osaki, Aiko Murai, Yoshihiko Hirohashi, Takanori Sasaki, Kazuhiro Ogi, Taka‐aki Tokura, Takayuki Kanaseki, Tomohide Tsukahara, Shinichiro Kina, Toshihiko Torigoe, Akihiro Miyazaki

**Affiliations:** ^1^ Department of Oral Surgery Sapporo Medical University School of Medicine Sapporo Hokkaido Japan; ^2^ Department of Pathology Sapporo Medical University School of Medicine Sapporo Hokkaido Japan; ^3^ Joint Research Center for Immunoproteogenomics Sapporo Medical University Sapporo Hokkaido Japan; ^4^ Center for Medical Education Gunma University Graduate School of Medicine Maebashi Gunma Japan; ^5^ Department of Pharmacology Graduate School of Medicine, University of the Ryukyus Ginowan Okinawa Japan

**Keywords:** cancer immunotherapy, CD44, peptide vaccine

## Abstract

Despite advancements in the treatment of oral cancer, cancer survival rates remain low, highlighting the need for new therapeutic strategies targeting cancer stem‐like cells. Cancer stem‐like cells are a small population of cancer cells within tumors that drive recurrence and metastasis. They are often resistant to conventional treatments. Immunotherapy has shown promise against cancer stem‐like cells, particularly with the use of cytotoxic T lymphocytes targeting specific markers. Survivin, an apoptosis protein inhibitor, is overexpressed in several malignancies, including oral cancer, and is associated with tumor recurrence and reduced survival. Survivin‐2B‐specific cytotoxic T lymphocytes were produced and evaluated for their ability to target CD44+ (cancer stem‐like cells) and CD44− cells (non‐cancer stem‐like cells), respectively, from oral cancer cell lines (HSC‐2 and HSC‐3, respectively). Quantitative polymerase chain reaction (qPCR) analysis confirmed similar survivin‐2B expression in both cell types. Cytotoxic T lymphocyte assays revealed the effective lysis of both cancer stem‐like cells and CD44− cell populations, supporting the potential of survivin‐2B‐specific cytotoxic T lymphocytes to overcome cancer stem‐like cell‐associated resistance. These findings suggest that survivin‐2B peptide vaccines are effective in preventing cancer relapse by targeting cancer stem‐like cells, with future directions aimed at developing multipeptide “cocktail” vaccines to reduce the risk of immune evasion.

## INTRODUCTION

Despite improvements in surgical techniques and chemotherapy, the prognosis for oral squamous cell carcinoma remains poor, with a 5‐year overall survival rate of only about 64.4% [1]. This highlights the urgent need to develop novel therapeutic strategies for oral cancers, particularly cancer stem‐like cells. Cancer stem‐like cells are believed to drive tumor initiation, progression, and recurrence [2]. Cancer stem‐like cells represent a small subset of cells within a tumor that possess self‐renewal capacity and the ability to give rise to heterogeneous lineages of cancer cells that make up the tumor. Furthermore, cancer stem‐like cells are often resistant to conventional chemotherapy and radiation therapy, which can lead to tumor recurrence following treatment [3, 4, 5]. Immunotherapy is considered to be effective for cancer stem‐like cells, and it has been reported that cancer stem‐like cells are targets for cytotoxic T lymphocyte immunotherapy [6]. Moreover, elimination of only cancer stem‐like cells subsets using cytotoxic T lymphocyte immunotherapy may be effective in controlling tumor formation in vivo [7]. There are many indicators of oral cancer malignancy other than cancer stem‐like cells, such as the degree of differentiation [8], nuclear morphology [9], specific genes (e.g., p53 mutations and VEGF/VEGFR expression) [10], metabolic capacity and spheroid morphology in 3D culture [11]. Among them, we considered cancer stem‐like cells to be the most viable and suitable therapeutic target. We have demonstrated previously that CD44 is a valuable marker useful for oral cancer stem‐like cell isolation [12].

Survivin is a characterized apoptosis protein inhibitor that is expressed abundantly in most solid and hematological malignancies [13]. However, it is barely detectable in normal tissues [14]. It has been demonstrated to increase tumor resistance to apoptotic stimuli, such as radiation and chemotherapy [15, 16]. Numerous studies have reported that the survivin gene is highly expressed in cancer cells and that its expression has prognostic value, being associated with increased tumor recurrence and reduced survival rates [17, 18, 19, 20]. A similar trend was observed for oral cancer [21, 22]. We previously investigated survivin and its splicing variants, survivin‐2B and survivin‐ΔEx3 [14]. Survivin expression is generally low in normal cells but elevated in cancer cells. We found that survivin‐2B and survivin‐ΔEx3 exhibit an even more restricted expression pattern, with lower levels in normal cells compared with survivin. We identified a novel HLA‐A24‐restricted T‐cell epitope, survivin‐2B80–88 (AYACNTSTL), derived from survivin‐2B. This peptide successfully induced cytotoxic T lymphocytes in HLA‐A24‐positive cancer patients, which effectively killed cancer cells expressing both HLA‐A24 and survivin‐2B [14]. The cytotoxic T lymphocyte specific for this peptide was successfully induced from peripheral blood mononuclear cells in 83% of HLA‐A24‐positive patients with colorectal cancer and exerted cytotoxicity against HLA‐A24‐positive/survivin‐positive adenocarcinoma cells [23]. Moreover, we reported previously that survivin‐2B peptide‐specific cytotoxic T lymphocyte was induced in 50% of HLA‐A24‐positive patients with oral cancer [24]. Based on these results, clinical trials on oral, colorectal, breast, and pancreatic cancers have been conducted using the survivin‐2B peptide [25, 26, 27, 28]. We have reported previously that the use of survivin‐2B peptide has been effective in patients with oral cancer [25]. However, the association between survivin‐2B and cancer stem‐like cells remains unclear. In this study, we elucidated how the survivin‐2B vaccine acts on oral cancer stem‐like cells.

## MATERIAL AND METHODS

### Cell culture

The human cell lines used were the following: oral carcinoma (HSC‐2 and HSC‐3), erythroleukemia (K562), and a TAP‐deficient T2 cell line stably expressing HLA‐A*24:02 (T2‐A24). Both HSC‐2 and HSC‐3 can be separated into cancer stem‐like cells and non‐cancer stem‐like cells [12]. Since the HLA‐A typing is HLA‐A24:02,33:02 in HSC‐2 and HLA‐A02:01,24:02 in HSC‐3 [29], they have the potential to be targeted by the survivin‐2B80–88 peptide, which is restricted to HLA‐A24. These two oral carcinoma cell lines were selected for this study for these reasons. K562 lacks functional HLA molecules and is commonly used as a negative control in cytotoxic T lymphocyte experiments [30]. K562 was purchased from ATCC, and HSC‐2 and HSC‐3 were purchased from the Japanese Cancer Research Resources Bank in Osaka, Japan. T2‐A24 was kindly provided by Dr K. Kuzushima (Aichi Cancer Center Research Institute). Upon arrival, the cell lines were immediately frozen in batches, with the culture period of each batch limited to a maximum of 8 weeks. For all experiments, low passage number cells were used (maximum 20 but more commonly 10 to 15 passages). The cells were cultured in Roswell Park Memorial Institute 1640 Medium or in Dulbecco's Modified Eagle Medium supplemented with 10% fetal bovine serum and 1% antibiotics and maintained at 37°C in a humidified atmosphere with 5% CO_2_.

### Magnetic sorting of the CD44+/− cells

The HSC‐2 and HSC‐3 cells were independently subjected to magnetic cell separation using CD44 (antihuman) MicroBeads (Miltenyi Biotec) according to the manufacturer's protocols. Previous studies have shown that the average purity after immunomagnetic bead enrichment was 26.03 ± 1.57%, 73.97 ± 1.56%, 24.95 ± 2.43% and 75.05 ± 2.43% for HSC2 CD44+, HSC2 CD44−, HSC3 CD44+ and HSC3 CD44−, respectively [12].

### Reverse transcription‐quantitative polymerase chain reaction (RT‐qPCR) for survivin‐2B expression

Total RNA was isolated from CD44+ and CD44− cells (HSC‐2 and HSC‐3) using TRIzol reagent (Invitrogen) and a Mini Kit (Qiagen) or an AllPrep deoxyribonucleic acid (DNA)/RNA Mini Kit (Qiagen) according to the manufacturer's protocol. The complementary deoxyribonucleic acid (cDNA) was synthesized via RT using SuperScript III and oligo (dT) primers (Life Technologies) and diluted for RT–quantitative polymerase chain reaction (qPCR) according to the manufacturer's protocol. cDNA amplification was performed using SYBR Green Master Mix (Thermo Fisher Scientific) for survivin‐2B and glyceraldehyde 3‐phosphate dehydrogenase (GAPDH) on a StepOne Real‐Time PCR System (Applied Biosystems). An initial denaturation step of 95°C for 10 min was followed by 50 cycles of denaturation at 95°C for 15 s and annealing/extension at 60°C for 60 s. GAPDH was used as the housekeeping gene. The cycle threshold (*C*_t_) values for each gene were corrected using the mean *C*_t_ value. mRNA levels were calculated using the ΔΔ*C*_t_ method and quantified using the 2^–ΔΔ^ *^C^* ^t^ method, normalized to the average *C*_t_ for the GAPDH gene expression levels. The primer pairs were as follows: survivin‐2B, 5'‐TCAAGGACCACCGCATCTCTAC‐3' and 5'‐GTGCTGGTATTACAGGCGTAAG‐3' (product size 221 bp), G3PDH, 5′‐ACCACAGTCCATGCCATCAC‐3' and 5'‐TCCACCACCCTGTTGCTGTA‐3′ (product size 452 bp).

### Synthetic peptide

The survivin‐2B80–88 (AYACNTSTL) peptide was designed using a previously described method [14]. The peptides were synthesized and purchased from Life Technologies. They were dissolved in DMSO and stored at −80°C before use.

### Cytotoxic T lymphocyte induction

The established method for cytotoxic T lymphocyte induction was employed [31]. Briefly, CD8+ cells were isolated from the peripheral blood mononuclear cells from “HLA‐A”24:02‐positive healthy donors using anti‐CD8 coupled to magnetic microbeads (Miltenyi Biotech). The remaining CD8− cells were phytohemagglutinin‐activated and used as phytohemagglutinin blasts [14]. The resulting phytohemagglutinin blasts served as potent antigen‐presenting cells [31]. CD8+ cells were cultured in AIM‐V medium (Life Technologies) containing L‐glutamine, 50 µg/mL streptomycin sulfate, 10 µg/mL gentamicin sulfate, 10% human serum (kind gift from Dr Takamoto, Japanese Red Cross Hokkaido Block Blood Center), 20 U/mL human Interleukin‐2 (IL‐2) (kind gift from Takeda Pharmaceutical) and 10 ng/mL IL7 (R&D Systems) for 4 weeks. CD8+ cells were repeatedly stimulated with autologous phytohemagglutinin‐blasts, which were activated from CD8− cells and pulsed with 20 mmol/L survivin‐2B80–88 peptide.

### Peptide–MHC tetramer assay and establishment of the cytotoxic T lymphocyte clone

To produce cytotoxic T lymphocyte clones, single cells that bind both anti‐CD8 (Beckman Coulter) and a survivin‐2B‐HLA‐A24 tetramer (MBL Life Science) were isolated using FACSAria II (BD Biosciences). They were expanded in AIM‐V medium containing 100 U/mL IL2, 1 mg/mL phytohemagglutinin, and x‐ray‐irradiated peripheral blood mononuclear cells from healthy volunteers.

### Enzyme‐linked immunospot interferon‐gamma assay

Cytotoxic T lymphocytes were added to enzyme‐linked immunospot (ELISPOT) plates coated with antihuman interferon‐gamma (IFN‐γ) (BD Biosciences) at 5.0 × 10^4^ cells per well. Then, T2‐A24 or the indicated cancer lines were placed in the corresponding wells (5.0 × 10^4^). T2‐A24 was preincubated with Survivin‐2B80–88 peptide [14] 20 mmol/L or DMSO (without peptide) at room temperature for 2 h. The wells were incubated with a biotinylated antihuman IFN‐γ antibody for 2 h at room temperature after incubation in a 5% CO_2_‐incubator at 37°C for 24 h, followed by the ELISPOT streptavidin–horseradish peroxidase antibody for 1 h. ELISPOT 3‐amino‐9‐ethyl carbazole Substrate Set (BD Biosciences) was used to visualize spots according to the manufacturer's protocol.

### Lactate dehydrogenase (LDH) cytotoxicity assay

A LDH cytotoxicity detection kit (Dojindo) was used to measure the LDH released from lysed target cells according to the manufacturer's protocol. The target cells (5.0 × 10^4^) were co‐incubated with the indicated cytotoxic T lymphocyte numbers (effector/target cell ratio) at 37°C for 24 h. The LDH released (indicating cytotoxicity) percentage was calculated as follows: % LDH released = 100 × (experimental LDH released − spontaneous LDH released)/(maximal LDH released − spontaneous LDH released). LDH values from cytotoxic T lymphocytes alone and target cells treated with lysis buffer (Dojindo) were used to estimate the spontaneous and maximum LDH releases, respectively.

### Statistical analysis

Data were analyzed via analysis of variance and Student's *t*‐test using spss version 23 (IBM) for comparisons between cell‐types in qPCR, ELISPOT, and LDH cytotoxicity assays. All analyses for these assays were performed with *n* = 3 per group. A *p*‐value ≤ 0.05 was considered to be statistically significant. Statistical analyses and graph assembly were performed using GraphPad Prism 9.5.0.

### Ethical approval

Ethical approval for this study was obtained from the Institutional Review Board of Sapporo Medical University School of Medicine (Approval Nos. 282‐134 and 322‐246).

## RESULTS

### Survivin‐2B gene expression in both CD44+/−  cells from oral cancer cell lines (HSC‐2 and HSC‐3)

**FIGURE 1:**
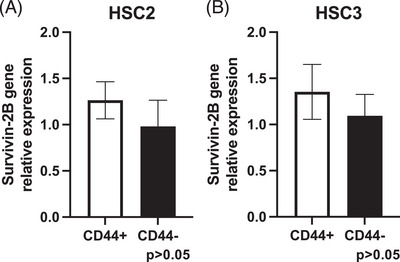
Both separated CD44+ and CD44− cells from oral cancer cell lines (HSC‐2 and HSC‐3) expressed the survivin‐2B gene. (A) The survivin‐2B mRNA expression on CD44+ cells from the HSC‐2 line are presented as a fold difference relative to CD44− cells from the HSC‐2 line. (B) The survivin‐2B mRNA expression on CD44+ cells from the HSC‐3 line are presented as a fold difference relative to CD44− cells from the HSC‐3 line. Each data point represents the mean of three independent experiments. The error bars denote the standard error of the mean (SEM). Data show no significant difference (*p* > 0.05).

qPCR analysis revealed that the survivin‐2B gene was expressed in both CD44+/− cells from the oral cancer cell lines (HSC‐2 and HSC‐3), with no significant difference observed between the two phenotypes (Figure 1A, B).

### Establishment of the survivin‐2B peptide‐specific cytotoxic T lymphocyte

**FIGURE 2:**
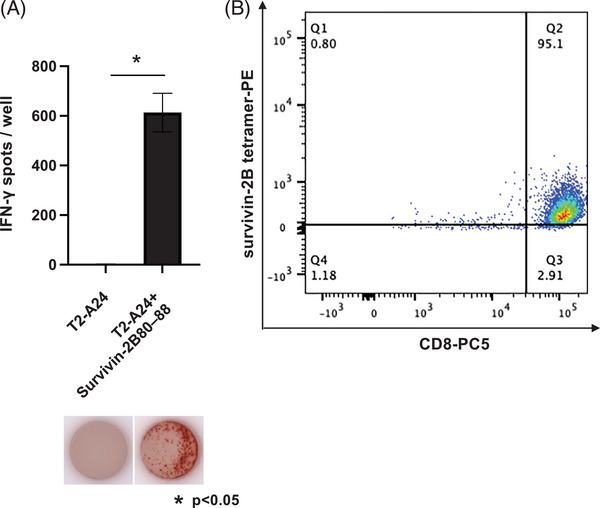
Cytotoxic T lymphocytes induced from the survivin‐2B‐derived synthetic peptide specifically lyses survivin‐2B80–88 peptide pulsed cells. (A) IFN‐γ expression on synthetic survivin‐2B80–88 peptide‐specific cytotoxic T lymphocytes as effector cells using T2‐A24 cells pulsed with synthetic survivin‐2B80–88 or nonpeptides as the target cell at a 1:1 ratio. Each data point represents the mean of three independent experiments. The error bars denote the standard error of the mean (SEM). ^*^ *p* < 0.05 (B) Flow cytometry of survivin‐2B‐specific CTLs stained with anti‐CD8 and a survivin‐2B‐HLA‐A24 tetramer.

Peripheral blood monocytes derived from donors were stimulated with survivin‐2B80–88 peptide to induce survivin‐2B‐reactive cytotoxic T lymphocytes. Additionally, to obtain cytotoxic T lymphocyte clones, single cells that were survivin‐2B‐HLA‐A24 tetramer‐positive and CD8+ were isolated with peptide–MHC tetramer assay. This study established several survivin‐2B80–88 peptide‐specific cytotoxic T lymphocyte clones, and one of them was selected as the “survivin‐2B‐specific cytotoxic T lymphocyte” based on the results of ELISPOT and tetramer assays. The clone chosen showed the most robust response in both assays, confirming its specificity for the survivin‐2B80–88 peptide. To confirm the survivin‐2B‐specific cytotoxic T lymphocyte specificity, an ELISPOT assay was performed. The survivin‐2B‐specific cytotoxic T lymphocyte showed a strong IFN‐γ response to the survivin‐2B80–88 peptide (Figure 2A). When the survivin‐2B‐specific cytotoxic T lymphocytes were stained with the survivin‐2B‐HLA‐A24 tetramer, a clear survivin‐2B‐HLA‐A24 tetramer‐positive cell population was confirmed (Figure 2B).

### Effect of survivin‐2B‐specific cytotoxic T lymphocyte on oral cancer stem‐like cells and non‐cancer stem‐like cells

**FIGURE 3:**
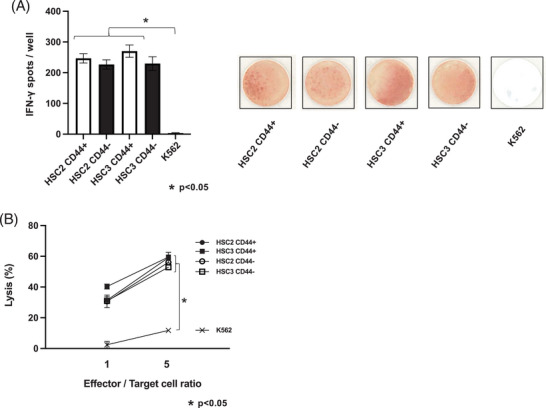
Cytotoxic T lymphocytes induced from the survivin‐2B‐derived synthetic peptide lyses CD44+/− oral cancer cells. (A) IFN‐γ expression on synthetic survivin‐2B80–88 peptide‐specific cytotoxic T lymphocytes as effector cells using CD44+/− cells from oral cancer cell lines (HSC‐2 and HSC‐3) and K562 as the target cells. Each data point represents the mean of three independent experiments. The error bars correspond to SEM. ^*^ *p* < 0.05. (B) LDH release cytotoxicity assay using synthetic survivin‐2B80–88 peptide‐specific cytotoxic T lymphocytes as effector cells and CD44+/− cells in HSC‐2 or HSC‐3 cells and K562 as the target cells at different ratios. Each data point represents the mean of three independent experiments. The error bars denote the standard error of the mean (SEM). ^*^ *p* < 0.05.

The ELISPOT assay on CD44+ and CD44− cells isolated from HSC‐2 and HSC‐3 co‐cultured with survivin‐2B‐specific cytotoxic T lymphocytes revealed that IFN‐γ, indicating cytotoxic T lymphocyte activation, was detected in both CD44+ and CD44− cells (Figure 3A). The LDH cytotoxicity assay on CD44+ and CD44− cells isolated from HSC‐2 and HSC‐3 co‐cultured with survivin‐2B‐specific cytotoxic T lymphocytes confirmed the level of cytotoxic T lymphocyte‐mediated killing, which induces apoptosis, in both CD44+ and CD44− cells (Figure 3B). In both the ELISPOT and LDH cytotoxicity assays, the responses of both CD44+ and CD44− cells were superior to those of the negative control, K562.

## DISCUSSION

Cancer stem‐like cells have been reported in head and neck cancers since 2007, and CD44 is one of the most well‐known cancer stem‐like cell isolation markers in head and neck cancers [32]. Furthermore, we have demonstrated previously that in oral cancer cells compared with CD44− cells, CD44+ cells have the characteristics of general cancer stem‐like cells, such as chemoresistance, increased expression of stem cell‐related genes, sphere‐forming ability, and a higher proportion of the G0/G1 phase in the cell cycle [12].

To date, the survivin‐2B80–88 peptide vaccine has exhibited a certain degree of therapeutic efficacy in HLA‐A24‐positive patients with oral, colorectal, breast, and pancreatic cancer [25, 26, 27, 28]. Most of these patients have failed to respond to standard treatments, and the vaccine has played a pivotal role as the last resort.

However, the survivin‐2B peptide vaccine has not been evaluated in cancer stem‐like cells, thus, we decided to evaluate it in this study.

The results of this study indicated that there was no significant difference in the gene expression level of the survivin‐2B gene between CD44+ and CD44− cells isolated from oral cancer cell lines. ELISPOT and LDH cytotoxicity assays revealed that the survivin‐2B‐specific cytotoxic T‐lymphocytes damaged the CD44+ and CD44− cells to the same extent as the gene expression results.

It has been reported previously that cytotoxic T lymphocytes induced by peptide vaccines are effective against cancer stem‐like cells [6, 7]. Considering this and the results of the present study, the survivin‐2B80–88 peptide can be considered one of the effective treatments for cancer stem‐like cells.

Moreover, considering that cancer stem‐like cells are the cause of relapse and metastasis, peptide vaccines against these cells are expected to be effective in preventing relapse. However, the possibility of immune evasion cannot be ruled out if the same peptide vaccine is used repeatedly. Thus, it would be desirable to have multiple peptides that are effective against cancer stem‐like cells. We will continue to develop peptide vaccines targeting cancer stem‐like cells so as to create a “cocktail vaccine” that combines multiple peptide vaccines.

## AUTHOR CONTRIBUTIONS

**Conceptualization**: Sho Miyamoto, Yoshihiko Hirohashi, Toshihiko Torigoe, and Akihiro Miyazaki. **Data curation**: Sho Miyamoto, Azuna Osaki, Aiko Murai, Takanori Sasaki, Kazuhiro Ogi, Takaaki Tokura and Shinichiro Kina. **Investigation**: Sho Miyamoto, Aiko Murai, Yoshihiko Hirohashi, Takayuki Kanaseki, Tomohide Tsukahara, Toshihiko Torigoe and Akihiro Miyazaki. **Writing—original draft**: Sho Miyamoto, Toshihiko Torigoe, Akihiro Miyazaki. **Writing—review & editing**: Sho Miyamoto, Yoshihiko Hirohashi, Toshihiko Torigoe, Shinichiro Kina, and Akihiro Miyazaki.

## CONFLICT OF INTEREST STATEMENT

The authors declare that they have read the article and have no competing interests.

## Data Availability

Raw data were generated at the Department of Oral Surgery, Sapporo Medical University School of Medicine. Derived data supporting the findings of this study are available from the corresponding author, SM, on request.
